# Emotional Intelligence and Adolescents’ Use of Artificial Intelligence: A Parent–Adolescent Study

**DOI:** 10.3390/bs15081142

**Published:** 2025-08-21

**Authors:** Marco Andrea Piombo, Sabina La Grutta, Maria Stella Epifanio, Gaetano Di Napoli, Cinzia Novara

**Affiliations:** Department of Psychology, Educational Science and Human Movement, University of Palermo, 90128 Palermo, Italy; sabina.lagrutta@unipa.it (S.L.G.); mariastella.epifanio@unipa.it (M.S.E.); gaetano.dinapoli@unipa.it (G.D.N.); cinzia.novara@unipa.it (C.N.)

**Keywords:** trait EI, adolescents, artificial intelligence, parenting styles, perceived social support, digital literacy, parental involvement

## Abstract

Artificial Intelligence (AI) profoundly shapes adolescents’ digital experiences, presenting both developmental opportunities and risks related to privacy and psychological well-being. This study investigates first the possible generational gap between adolescents and their parents in AI use and trust, and then the associations between the Trait Emotional Intelligence (trait EI), parenting styles, perceived social support, and parental involvement on adolescents’ use and trust in AI-based technologies. Participants were 170 adolescents (aged 13–17) and 175 parents from southern Italy, who completed standardized questionnaires assessing parenting styles, Trait Emotional Intelligence (Trait EI), social support, digital literacy, and use and trust in AI. Adolescents used AI more frequently than parents, especially for school- or work-related support and were more likely to seek behavioral advice from AI. They also showed higher trust in AI data security and the quality of behavioral advice than parents. Moreover, greater trait EI and more authoritative (vs. authoritarian) parenting were associated with less frequent AI use and lower use and trust in AI. In 47 matched parent–adolescent dyads, cluster analysis identified Balanced Users (higher trait EI, authoritative parenting, stronger support, cautious AI use) and At-Risk Users (lower trait EI, authoritarian parenting, lower support, heavier and more trusting AI use) Despite no causal inferences can be drawn due to the correlational nature of the data, the results suggested the importance of considering adolescents’ trait EI and authoritative parenting practices in supporting balanced and critical digital engagement, highlighting the concept of a “digital secure base” as essential for navigating the evolving digital landscape.

## 1. Introduction

Artificial Intelligence (AI) has revolutionized many aspects of contemporary society, significantly influencing the lives of minors in terms of safety and privacy, as well as communication between parents and children (Basso, 2023). In this sense, it is redefining the way they interact with technology, acquire new information, and build their social identity (Lai et al., 2023). Decades of work on trust in socio-technical systems show that young users’ attitudes toward novel technologies evolve through repeated interaction and continuous “calibration” of expectations and outcomes (de Visser et al., 2023; Hancock et al., 2011). In particular, adolescents represent a population group particularly exposed to the effects of such innovations, as they are at a particularly delicate stage of emotional and cognitive development. The increasing use of AI-based tools in educational platforms, social media, and digital communication systems has opened up new opportunities for improving learning and social interaction, but it has also raised various concerns about safety, privacy, and the impact on psychological well-being.

A central aspect of the discussion concerns how adolescents acquire, use, and develop digital skills that enable them to use AI responsibly and consciously. Consistent with European digital-competence frameworks, digital literacy is conceived broadly, spanning information/data literacy, communication and collaboration, safety and privacy, and problem solving, competencies about digital devices that are directly implicated when adolescents interact with AI-mediated tools (Vuorikari et al., 2022). In this sense, it is important to take into consideration the possible generational gaps in the knowledge and use of digital devices and AI systems between parents and adolescents. For instance, parents with strong digital literacy and a calibrated (rather than unquestioning) trust in AI are better equipped to recognize both the benefits and the potential hazards of AI-based activities. This competence enables them to be more involved and model critical engagement, more informed digital contexts for their adolescents (Celik, 2023). However, the growing body of existing literature suggests that the process of digital literacy depends not only on access to technologies but also on a range of individual, family, and social factors (Kozak & Fel, 2024; Dang & Li, 2025). In particular, some studies have highlighted the crucial role of parenting styles, emotional intelligence, and perceived social support in regulating online behaviors, as well as in the ability to make informed decisions in the digital environment (Livingstone & Helsper, 2008; Gruchel et al., 2022). In line with this evidence, parenting style has also been linked to adolescents’ problematic Internet use through regulatory emotional self-efficacy, highlighting the regulatory pathway by which family climate shapes online behavior (Chen et al., 2021).

More generally, parenting styles represent a key variable in the digital socialization process. According to the Baumrind model (Baumrind, 1967) and subsequent revisions (Mccoby & Martin, 1983), they are distinguished into four main categories: authoritative, authoritarian, permissive, and neglectful. An authoritative style, characterized by high levels of support and monitoring, may be associated with the better development of social and emotional skills in adolescents, with a positive impact on their ability to manage digital interactions safely and in a balanced manner. Conversely, an authoritarian style, characterized by rigid control and limited communication, may lead to less decision-making autonomy and dysfunctional use of technology. Another important variable in adolescent functioning is emotion regulation, which plays a key role in how individuals respond to digital challenges. From an attachment perspective, emotional regulation is first scaffolded in infancy within the primary caregiver relationship. Through attuned responses, need fulfillment, and the co-construction of meaning around affective experiences, caregivers provide a secure base that enables children to explore and gradually internalize self-regulatory skills (Bowlby, 1988). In this sense, the attachment theory offers a complementary lens: when caregivers provide a reliable “secure base” that couples emotional availability with guidance, whereby children and adolescents feel supported in exploring both physical and, increasingly, digital environments (Bowlby, 1988; Lancini, 2019). Accordingly, authoritative parenting can therefore be viewed as a behavioral expression of this secure-base function, whereas authoritarian or unresponsive family relational context may leave young people seeking regulatory support from technological agents instead of human relationships (Lancini & Turuani, 2020; Boerchi et al., 2020). In line with this, parenting style has been shown to influence adolescents’ ability to understand and regulate emotions, with more supportive and responsive approaches fostering stronger emotional competencies (Argyriou et al., 2016).

Among the wide landscape of emotional competencies, one construct that has gained increasing attention is Trait Emotional Intelligence (Trait EI), defined as a set of emotional self-perceptions related to how individuals experience and manage emotions in everyday life (Petrides et al., 2007), including also personal dispositions such as empathy, emotion regulation, and self-control.

There is mounting evidence that trait EI is influenced by early family interactions and parenting practices. Adolescents exposed to authoritative parenting, characterized by warmth, monitoring, and open dialogue, tend to exhibit higher levels of trait EI, which in turn supports adaptive emotional regulation (Argyriou et al., 2016). In the context of digital environments, trait EI is particularly relevant. Studies have shown that adolescents often turn to digital devices not only for communication or entertainment but also as external regulators of emotion—tools used to cope with stress or modulate affective states (Granow et al., 2020). Moreover, recent studies have shown that trait EI serves as a protective factor in adolescence, reducing psychological vulnerability (Mikolajczak et al., 2009; Zhang et al., 2022) and also mediating the impact of social media use, thereby enhancing the buffering effect of perceived social support (Riolo et al., 2025).

In this sense, adolescents with higher trait EI may be better equipped to regulate their emotional responses in terms of both positive and negative affect (Zhao et al., 2020), without excessive reliance on technology, thereby promoting a more balanced and intentional use of AI-powered systems.

Although trait EI is widely recognized as influencing emotional regulation and interpersonal interactions, the theoretical connection between trait EI and trust toward AI requires further elaboration. According to contemporary human–machine trust models (Glikson & Woolley, 2020), trust in AI comprises two main dimensions: a cognitive dimension, which involves perceived competence and reliability of AI, and an affective dimension, relating to perceived emotional and relational alignment between humans and machines. Individuals with higher trait EI are generally more adept at managing their emotional states and interpreting interpersonal cues (Petrides et al., 2016a). Thus, they may exhibit greater caution and critical evaluation when interacting with AI, particularly regarding AI’s ability to provide empathetic and emotionally appropriate responses. Recent developmental work indicates that adolescents utilize AI-mediated tools for a wide range of purposes, including academic support, creative production, mental health queries, and relationship advice, and that heavier use often coincides with reliance on the system’s perceived competence and benevolence (Glikson & Woolley, 2020; Klarin et al., 2024; Xia et al., 2023). In this sense, trait EI may serve as an important psychological factor shaping adolescents’ expectations and reliance on AI systems, influencing both their perceived credibility of AI-provided information and their willingness to engage emotionally with AI.

Another factor to consider is perceived social support, which could play a significant role in moderating the impact on adolescents’ lives. Generally, the support from the primary network may somehow facilitate greater resilience to digital challenges and promote more responsible behavior in the use of advanced technologies (Wentzel, 1998). In particular, parental involvement in their children’s digital education is crucial for promoting a balanced approach to AI management through active mediation strategies that encourage conscious and critical use of technology. Moreover, how adolescents perceive community support can significantly impact their online experiences: those who feel socially supported may use social networks to strengthen their relationships, while those with inadequate social support may develop a dependency on digital interactions to compensate for this lack (Novara et al., 2025). However, despite growing interest in how adolescents engage with digital environments, current literature remains fragmented in integrating personal, social, familial, and technological variables within a unified framework. Most studies have examined these dimensions in isolation—focusing either on parenting style, digital literacy, or emotional competencies—without exploring how these factors interact in shaping adolescents’ trust and responsible use of AI technologies. Additionally, while some research has addressed digital literacy, few have distinguished between adolescents’ and parents’ digital competencies or examined how parental engagement in AI-based systems influences youth behavior and attitudes. Moreover, the construct of trust in AI, a central psychological mechanism for navigating digital ecosystems, has been largely overlooked in developmental studies, particularly in relation to emotional traits such as Trait Emotional Intelligence or perceived social support. To our knowledge, no prior research has simultaneously investigated the roles of parenting styles, digital literacy, and AI usage patterns in both adolescents and parents, as well as individual emotional resources such as trait EI and perceived social support, in predicting adolescents’ relationships with AI.

### The Present Study

The aim of the research, conducted as part of the PRIN project “*Children as Vulnerable Users of IoT and AI-Based Technologies: A Multi-Level and Interdisciplinary Approach—CURA*”, was to thoroughly analyze how the personal, familial, and social factors influence adolescents’ ability to navigate the AI-driven digital landscape. Therefore, the study aims to investigate first, a possible generational gap in use and trust in AI-based technologies between parents and adolescents, secondly, how the relationship between personal, familial, and social variables can be associated with the development of strong digital literacy and more effective management of the risks and opportunities presented by artificial intelligence. According to these objectives, three main research questions and hypotheses:

1. Do adolescents and parents differ in reported AI use and trust? We anticipated that adolescents would report greater use and higher trust than parents, reflecting generational differences in digital familiarity.
2. Are adolescents’ trait EI, parenting style, perceived family support, and parental digital involvement associated with their AI use and trust in AI? We expected higher trait EI and more authoritative parenting to be associated with more cautious AI engagement and lower trust; authoritarian parenting was expected to be associated with heavier use and more trust in AI.
3. When considering matched parent–adolescent dyads, is it possible to detect patterns across emotional (trait EI), relational (parenting, family support), and AI use and trust variables clustering adolescents into distinct risk vs. balance profiles?

## 2. Materials and Methods

### 2.1. Participants and Procedures

The study involved a total sample of 345 participants: 170 adolescents (aged 13–17 years, M = 15.2, SD = 1.3) and 175 parents (Mean age = 49.22, SD = 8.91; 144 mothers and 31 fathers). An a priori power analysis was conducted using G*Power 3.1 (Faul et al., 2009) to determine the minimum sample size required to detect a medium effect size (f = 0.25) with α = 0.05 and power (1 − β) = 0.80 in between-group comparisons. The analysis indicated a minimum of 128 participants per group, suggesting that the obtained sample size was adequate for the planned statistical comparisons. Participants were recruited through local secondary schools and community groups, primarily located in southern Italy. Additionally, a matched subsample consisting of 47 parent-adolescent pairs was identified and used for more detailed analyses.

Data collection was conducted via online structured questionnaires administered through the Qualtrics platform. Parents and adolescents completed separate questionnaires, ensuring the confidentiality and privacy of responses. Before participation, informed consent was obtained from both adolescents and their parents, following a comprehensive explanation of the study’s objectives and procedures.

All procedures complied with the ethical standards of the relevant national and institutional committees on human experimentation and with the Declaration of Helsinki of 1975 (revised in 2008). The study was approved by the Bioethics Committee of the institution of the first author (n.180/2023).

### 2.2. Measures

#### 2.2.1. Parenting Style

Parenting styles were assessed using the Parenting Styles and Dimensions Questionnaire (PSDQ) developed by Robinson et al. (2001). The PSDQ is a widely used self-report instrument designed to evaluate parenting behaviors based on Baumrind’s theoretical model, identifying three core parenting styles: authoritative, authoritarian, and permissive. Each style includes multiple sub-dimensions reflecting specific parenting practices. In the Italian context, research has shown some culturally specific interpretations of parenting behaviors. In particular, practices typically labeled as permissive (e.g., involving children in decision-making) are not perceived as a distinct parenting style, but rather as a core component of the authoritative style.

This was confirmed by Tagliabue et al. (2018), who found that in Italian adolescent samples, factor structures revealed a strong overlap between permissive elements and authoritative dimensions. As such, the authoritative style in this study is interpreted broadly to include democratic behaviors. Recent research confirms its reliability and validity in different cultural settings, and the reliability of this study was good: Cronbach’s alpha = 0.85.

#### 2.2.2. Perceived Social Support

The Multidimensional Scale of Perceived Social Support (MSPSS; Zimet et al., 1988) was used to assess perceived social support. It is a 12-item scale that measures perceived social support from three sources: Family, friends, and significant others. The scale was rated on a seven-point Likert response format (1 = “very strongly disagree” to 7 = “very strongly agree”). The total scores range from 12 to 84, with higher scores indicating greater total perceived social support from all three sources. The internal reliability of the scale in our sample was good: Cronbach’s alpha = 0.89.

#### 2.2.3. Trait Emotional Intelligence

The Trait Emotional Intelligence Questionnaire—Adolescent Short Form (TEIQue-ASF; Petrides et al., 2016b) and the Italian version of the Trait Emotional Intelligence Questionnaire-360 Short Form (TEIQue-360-SF; Petrides, 2009) were used to measure adolescents’ perceived trait EI and parents’ ratings of their trait EI, respectively. Both versions comprise 30 short statements on a 7-point Likert scale designed to measure global trait EI and the four broad factors of trait EI: Well-being, Self-Control, Emotionality, and Sociability. For the purpose of this study, only global trait EI scores were used, and the questionnaires showed good internal reliability, both for parents’ rating version (Cronbach’s alpha = 0.92) and for adolescents’ self-reported version(Cronbach’s alpha = 0.90).

#### 2.2.4. Digital Literacy

The digital literacy was measured using the Digital Literacy Scale for Teenagers, developed and validated by Rodríguez-de-Dios et al. (2016). This scale assesses adolescents’ competence in navigating and understanding digital media environments through a multidimensional framework.

The questionnaire includes four key dimensions, such as Technological Skills—ability to use digital tools and platforms (e.g., search engines, devices). Information Skills—evaluating, selecting, and managing online information. Critical Understanding—awareness of media biases, persuasive content, and risks in digital communication. Digital Participation—responsible engagement in online communities and content creation.

The scale has demonstrated strong psychometric properties in adolescent populations, including good internal consistency across all subscales (α > 0.80). It is particularly relevant for measuring both functional and reflective components of digital literacy in young users, making it a robust tool for studies investigating technology use, digital citizenship, and online behavior in youth. The reliability for the global score was good for this study (Cronbach’s alpha = 0.87).

#### 2.2.5. The Parental Involvement in Internet Use

Parental involvement was measured using the scale developed by Gruchel et al. (2022), assessing various aspects of parental involvement in their children’s internet use, including parental instruction, co-use, and mediation strategies. Parents responded to items such as “I help my child search for information online” and “I discuss internet safety with my child” on a 5-point Likert scale from 1 (never) to 5 (always). The reliability for this study was good (Cronbach’s alpha = 0.90).

#### 2.2.6. Usage and Trust in AI

Participants’ behaviors regarding AI usage were assessed through a specifically developed set of four items designed to capture the frequency and nature of their interactions with AI-powered systems. Adolescents and parents rated their engagement with AI using a 5-point Likert scale ranging from 1 (never) to 5 (very often). The items specifically addressed the following behaviors: “I share personal data with AI software”, “I ask AI software for advice on how to behave in certain situations”, “I seek general cultural information using AI software”, and “I use AI software as support in my schoolwork/professional tasks”. Higher scores on this scale indicated more frequent interaction with AI-based technologies.

Additionally, participants’ trust in AI was evaluated through five items explicitly developed to reflect the multidimensional nature of human-machine trust as suggested by Glikson and Woolley (2020) in a recent meta-analysis. These items reflect dimensions such as the perceived reliability, transparency, and benevolence in terms of relational support. The respondents indicated their agreement with each statement using a 5-point Likert scale ranging from 1 (never) to 5 (very often). The items measuring reliability and transparency included statements that reflected the system’s technical soundness, accuracy, and data security, including “I think the data I provide to AI software are safe”, “I believe the information provided by AI software is truthful and accurate”, and “I think AI software can perform certain tasks better than humans”, while, for benevolence in terms of relational support, items reflected the perception that the system can offer helpful guidance compared with close others, including “I think AI software can provide better advice than members of my family” and “I think AI software can provide better advice than my friends”. Higher scores indicated greater trust in the capabilities, accuracy, and security offered by AI-based technologies. Furthermore, exploratory factor analysis (EFA) with varimax rotation confirmed the multidimensional structure of both Use and Trust in AI scales, identifying two distinct factors for each: Information Reliability (factor loadings = 0.71–0.75) and Relational Support/Behavioral Advice (factor loadings = 0.70–0.96). Together, these two factors explained 60% of the total variance, thus providing preliminary evidence for the construct validity of these measures for this study.

### 2.3. Statistical Analysis

All statistical analyses were conducted using IBM SPSS Statistics (Version 25).

Descriptive statistics, including means and standard deviations for continuous variables, as well as frequencies and percentages for categorical responses, were computed to summarize demographic information and the distribution of AI-related behaviors across the adolescent and parent samples.

Inferential analyses involved a two-way Analysis of Variance (ANOVA) to examine mean-level differences between adolescents and their parents regarding trust in AI, Trait Emotional Intelligence, perceived social support, and frequency of AI usage behaviors. Differences in categorical variables related to AI usage were explored using Chi-square tests of independence, which allowed us to identify significant generational gaps in AI attitudes and behaviors.

Pearson’s correlation coefficients were calculated to investigate the bivariate associations among the variables.

Finally, a two-step cluster analysis was conducted on the matched parent-adolescent dataset to identify distinct adolescent profiles in terms of their emotional competencies, perceived social support, parenting styles, and patterns of AI engagement. We selected Two-Step Cluster Analysis as an appropriate exploratory method to identify naturally occurring homogeneous subgroups (clusters) within our sample. Different from other clustering methods (e.g., hierarchical or K-means cluster analysis), Two-Step Cluster Analysis offers several methodological advantages relevant to our research aims, such as the ability to handle both continuous and categorical variables, the automatic determination of the optimal number of clusters, and the robustness with moderate to small sample sizes. This approach enabled the classification of adolescents into clearly interpretable clusters representing “at-risk” versus “not-at-risk” profiles concerning problematic AI usage, thus enhancing the interpretability and applicability of the study’s findings. For all statistical tests, a two-tailed significance threshold was set at *p* < 0.05.

## 3. Results

### 3.1. Descriptive Statistics and Differences Between Parents and Adolescents

Descriptive statistics and differences between parents and adolescents for the study variables are reported in Table 1.

Descriptive analyses revealed significant differences between adolescents and their parents in terms of their use of AI-based software and trust in AI. Specifically, adolescents reported a significantly greater frequency of using AI for school or work support compared to parents (*χ*^2^ = 20.428, *p* < 0.001). For instance, approximately 32% of adolescents used AI software for these purposes “often” or “very often”, versus only 17% of parents. Significant differences were also observed regarding seeking behavioral advice from AI systems (*χ*^2^ = 11.788, *p* = 0.019): adolescents were more inclined (22%) compared to their parents (12%) to occasionally or frequently seek advice from AI. Conversely, sharing personal data with AI showed a marginal difference approaching significance (*χ*^2^ = 9.367, *p* = 0.053), with adolescents slightly more likely than parents to share personal information with AI systems (30% vs. 18%, respectively). Regarding trust toward AI, substantial differences emerged between the two groups. Adolescents demonstrated significantly higher trust concerning data security (*χ*^2^ = 29.010, *p* < 0.001), with 15% of adolescents often or very often believing that their data were secure, compared to only 18% of parents who agreed frequently. Moreover, adolescents strongly believed that AI could provide better advice than their family (*χ*^2^ = 12.683, *p* = 0.013). A similar pattern emerged regarding the perceived accuracy of information provided by AI (*χ*^2^ = 11.812, *p* = 0.019), with adolescents being more likely to trust AI-generated information compared to their parents. Effect sizes (Cramer’s V) and their 95 % confidence intervals are reported in Table 1 for all *χ*^2^ comparisons between adolescents and parents. Values ranged from 0.14 to 0.29, corresponding to small-to-medium magnitudes.

### 3.2. Correlational Analysis

Bivariate associations between variables are reported in Table 2. Specifically, an authoritative parenting style positively correlated with trait EI *(r* = 0.35, *p* < 0.001) and digital literacy (*r* = 0.33, *p* < 0.001). Conversely, an authoritarian parenting style negatively correlated with Trait EI (*r* = −0.31, *p* < 0.001) and positively correlated with problematic AI behaviors, such as frequently sharing personal data with AI (*r* = 0.23, *p* = 0.01) and seeking behavioral advice from AI (*r* = 0.34, *p* < 0.001).

As regards trait EI, it showed a negative correlation with several items of AI usage. Higher trait EI was associated with lower frequencies of requesting behavioral advice from AI (*r* = −0.17, *p* < 0.001), using AI for school or work-related tasks (r = −0.11, *p* < 0.05), and lower trust in AI capabilities, such as believing AI provides superior advice compared to family members (*r* = −0.14, *p* = 0.01) or friends (r = −0.15, *p* = 0.01).

Furthermore, parental involvement in digital activities positively correlated with increased AI usage behaviors, including sharing personal data (*r* = 0.28, *p* < 0.001) and seeking AI-based behavioral advice (*r* = 0.27, *p* < 0.001). Digital literacy also positively correlated with several AI behaviors, including using AI for information gathering (*r* = 0.22, *p* < 0.001) and school or work-related support (*r* = 0.24, *p* < 0.001).

### 3.3. Identifying Adolescents’ Profiles Related to AI Usage: A Preliminary Cluster Analysis

A Two-Step cluster analysis was conducted to identify distinct profiles of adolescents (n = 47 dyads, representing matched parent-adolescent pairs) based on their emotional intelligence (trait EI), parenting styles, digital behaviors, trust in AI, and perceived social support. A two-cluster solution was specified and yielded fair cluster quality according to the silhouette measure of cohesion and separation = 0.4, indicating an acceptable degree of differentiation between groups despite the relatively small sample size. The analysis revealed two clearly distinguishable clusters:

Cluster 1 (Balanced users, 62% of the sample): Adolescents in this cluster demonstrated higher levels of authoritative parenting style (Mean = 4.16, SD = 0.44), higher trait EI scores (Mean = 4.87, SD = 0.71), and greater perceived family support (Mean = 24.29, SD = 3.74). Moreover, these adolescents reported relatively lower levels of authoritarian parenting (Mean = 1.93, SD = 0.40), and lower levels of some AI-related behaviors such as sharing personal data (Mean = 1.58, SD = 0.81) and seeking behavioral advice from AI (Mean = 1.23, SD = 0.50). Trust toward AI was cautious in this cluster, with adolescents expressing relatively low agreement with statements suggesting AI superiority compared to parents (Mean = 1.45, SD = 0.96) or friends (Mean = 1.58, SD = 0.56).

Cluster 2 (At-risk users, 38% of the sample): representing approximately one-third of the adolescents, this cluster indicated more problematic patterns in AI use and psychological functioning. Adolescents in this group were characterized by higher levels of authoritarian parenting (Mean = 2.30, SD = 0.55), lower authoritative parenting scores (Mean = 3.70, SD = 0.84), and significantly lower trait EI (Mean = 4.30, SD = 0.64). Additionally, adolescents within this cluster exhibited markedly higher engagement in AI-related behaviors, such as sharing personal data (Mean = 2.63, SD = 1.31), actively seeking behavioral advice from AI (Mean = 2.81, SD = 1.33), and relying more intensively on AI for academic tasks (Mean = 3.44, SD = 1.32). They also demonstrated notably higher trust in AI, agreeing more strongly that AI provides better advice than parents (Mean = 2.94, SD = 0.96) and friends (Mean = 3.06, SD = 1.18), and is more accurate (Mean = 3.38, SD = 1.06). This group also reported substantially lower perceived family support (Mean = 18.31, SD = 5.64). All the results discussed above are reported in Table 3.

## 4. Discussion

The current study explored adolescents’ relationships with Artificial Intelligence (AI), first examining differences in the use and trust of AI with their parents, and secondly focusing on the associations with trait emotional intelligence (trait EI), parenting styles, perceived social support, and parental involvement. We specifically examined adolescents’ and parents’ engagement with AI, both in terms of practical usage behaviors and attitudinal trust in AI-driven systems. Our findings outline a complex pattern of associations between these variables and adolescents’ AI interactions. First, the differential patterns observed in adolescents’ practical AI usage, such as sharing personal data, seeking behavioral guidance, and employing AI for educational tasks, suggest some important psychological dimensions driving digital behaviors. Trait EI emerged as one of the most closely related factors, showing a significant negative association with all aspects of AI usage and trust. This may reflect that adolescents with higher trait EI are less likely to share sensitive information and are less dependent on AI for emotional or social advice. The significant negative association between trait EI and trust in AI could suggest a role of emotional intelligence: adolescents with higher EI could tend to be more cautious and critically engaged with digital content, making informed decisions rather than relying blindly on AI recommendations (Argyriou et al., 2016). This result aligns with recent research indicating that emotionally intelligent individuals are typically more skilled in critical thinking and emotional self-regulation, and are therefore less prone to externalizing emotional regulation via digital means (Zhang et al., 2022; Riolo et al., 2025). In contrast, adolescents with lower trait EI may perceive AI systems as immediate, efficient, and unbiased sources of support, potentially compensating for emotional vulnerabilities and difficulties in offline interpersonal relationships (Granow et al., 2020; Petrides et al., 2016a). The correlation of parenting style with AI-related behaviors was also evident. Authoritative parenting, characterized by warmth, dialogue, and appropriate boundaries, is associated with more critical digital skills and emotional autonomy, which may reduce adolescents’ uncritical dependency on AI. This parenting style enhances digital literacy, promotes cautious engagement, and cultivates skepticism towards blindly accepting AI-generated information (Chen et al., 2021; Gruchel et al., 2022). Conversely, authoritarian parenting, defined by rigid, restrictive, and less communicative approaches, is significantly associated with adolescents’ trust in AI, especially regarding behavioral advice, and engaging in potentially risky AI behaviors. Adolescents experiencing authoritarian parenting may rely more heavily on AI systems for advice and guidance due to limited communication or inadequate emotional support from their parents, creating a greater dependency on technology as a substitute for parental and peer interactions (Boerchi et al., 2020; Chen et al., 2021). Moreover, regarding parental involvement in digital activities, higher parental involvement was positively correlated with certain forms of AI usage, such as academic support and data sharing. While parental involvement is typically regarded as protective, our data suggest a complexity in how it operates: parental involvement without emotional warmth or clear authoritative guidance might inadvertently foster uncritical acceptance of digital tools, amplifying potential risks (Gruchel et al., 2022). Indeed, previous research has highlighted that active parental mediation must be carefully balanced with promoting adolescents’ autonomy and critical digital skills; otherwise, excessive involvement without appropriate emotional support can paradoxically encourage overreliance on digital and AI-driven systems (Livingstone & Helsper, 2010; Hernandez et al., 2024). Regarding trust towards AI, our results demonstrated clear generational gaps, with adolescents displaying significantly greater trust in AI’s data security, information accuracy, and advisory capabilities than their parents. Adolescents’ higher trust may reflect generational familiarity and comfort with digital environments; however, this trust can expose them to significant vulnerabilities if not mediated by adequate emotional competencies and critical thinking skills (Riolo et al., 2025). Despite adolescents’ higher confidence in AI, such systems do not provide the co-regulatory, containing functions of human caregiving; they can offer information or prompts, but cannot replace the relational attunement required for emotional regulation. Importantly, emerging international evidence indicates that baseline trust in AI varies across cultural settings and sociodemographic groups, for example, comparative work with students in Poland and the United Kingdom and recent global reviews documenting cultural modulation of factors such as transparency, anthropomorphism, and perceived capability (Kozak & Fel, 2024; Dang & Li, 2025) while longitudinal research in Asian youth samples further suggests that motivations for AI use (e.g., socio-emotional support) shape downstream adjustment (Huang et al., 2024).

Finally, our cluster analysis provided additional depth to these interpretations by distinguishing two distinct adolescent profiles. The “At-risk users” cluster was marked not only by higher trust in AI and intensive usage but also by lower trait EI, authoritarian parenting practices, and lower family perceived social support. Conversely, the “Balanced users” cluster exhibited lower trust, balanced AI usage, higher Trait EI, authoritative parenting practices, and stronger perceived support. These clusters illustrate the complexity of adolescents’ relationships with AI, demonstrating how emotional, familial, and social factors interact to create either protective or risk-enhancing contexts (Granow et al., 2020; Chen et al., 2021). In particular, the co-occurrence of heavier and more trusting AI engagement with lower trait EI and more authoritarian contexts is consistent with turning to technological agents when relational support is constrained—further underscoring the need to strengthen human scaffolding rather than expecting technology to deliver emotional containment. However, because the cluster solution is preliminary and based on a relatively small matched parent–adolescent subsample (n = 47), this interpretation warrants replication in larger samples before firm conclusions can be drawn.

Building on these considerations, interventions aimed at fostering healthier relationships with AI should incorporate emotional education programs that focus explicitly on enhancing adolescents’ emotional intelligence. School-based and family-oriented interventions should emphasize authoritative parenting strategies, encouraging parents to offer emotional warmth, engage in open dialogue, and establish clear digital boundaries. Furthermore, parental digital mediation must be balanced, ensuring active guidance while supporting adolescents’ autonomy and the development of critical digital skills (Vuorikari et al., 2022). The current study’s findings clearly indicate that family relational factors, specifically, parenting styles and parental involvement, are significantly related to adolescents’ interactions with AI technologies. Our findings indicate that, just as young children require a secure emotional base in early childhood to safely explore and learn from their physical environment (Bowlby, 1988), adolescents similarly require a “digital secure base” from which they can confidently navigate and explore an increasingly complex and rapidly evolving digital landscape. The concept of a digital secure base is built upon Bowlby’s attachment theory and emphasizes the need for a stable, supportive, and emotionally responsive caregiving context that enables adolescents to internalize regulatory mechanisms, thereby allowing safe and balanced digital exploration (Lancini, 2019; Lancini & Turuani, 2020). Here, the base remains relational; the secure base remains the human caregiving relationship (warm, containing, and regulatory), while the modifier ‘digital’ refers to the online and AI-mediated environments that adolescents increasingly navigate for exploration.

From this perspective, effective interventions aimed at mitigating risks associated with digital engagement should prioritize strengthening familial relational dynamics and parents’ digital literacy to foster a robust digital secure base. This would entail educational initiatives directed toward parents, emphasizing the importance of adopting authoritative practices that support autonomy while simultaneously offering emotional availability and clear boundaries. As argued by Lancini (2019), promoting such a secure relational context does not simply limit adolescents’ digital interactions but instead provides them with essential emotional scaffolding, fostering resilience, critical thinking, and emotional regulation, which are skills crucial for navigating digital environments autonomously and responsibly.

Consequently, our results support the need for interventions aligned with attachment-informed approaches (Lancini & Turuani, 2020), recognizing family relationships as the cornerstone of healthy digital exploration and integration. Specifically, these patterns suggest that AI heightens, rather than reduces, the developmental need for human co-regulation; technological systems cannot provide the emotional containment that caregiving relationships offer. However, the applied implications are offered as hypothesis-generating targets for future longitudinal and experimental work, rather than demonstrated causal pathways. Future research should continue to explore these relational dimensions within broader developmental frameworks, reinforcing the central role of emotional security in preparing adolescents to face the ongoing challenges posed by rapid technological evolution.

### Limitations and Future Directions

Despite the significant contributions of this study to understanding the interplay of emotional, familial, and social factors influencing adolescents’ engagement with AI, several limitations should be acknowledged. First, the cross-sectional design precludes establishing causal relationships among variables. Although our findings suggest clear associations, longitudinal studies are needed to clarify how parenting styles, emotional intelligence, and social support dynamically influence adolescents’ evolving relationship with digital and AI-driven technologies over time. Additionally, the relatively small sample size in the matched parent-adolescent dyads (N = 47) limits the generalizability and statistical robustness of the cluster analysis. Future studies should utilize larger and more diverse samples to confirm the stability and replicability of these identified profiles.

Another limitation pertains to reliance exclusively on self-report measures, which are subject to social desirability bias and might not fully capture actual digital behaviors and parental practices, raising the possibility of common-method bias. We took two precautionary steps to mitigate this risk: (a) questionnaires were administered under strict guarantees of anonymity and confidentiality, reducing social-desirability pressure; and (b) different informants supplied information on several key constructs (e.g., parents reported on parenting style and involvement, whereas adolescents reported on AI trust/usage and trait EI). Moreover, the study primarily focused on psychological and relational determinants, potentially overlooking broader contextual factors such as socio-economic status, cultural differences, and digital inequalities, which can significantly shape adolescents’ AI-related behaviors and attitudes.

Future studies should incorporate multiple assessment strategies, including observational methods, digital-usage logs, and qualitative interviews, to triangulate findings and further reduce these limitations. Additionally, longitudinal intervention studies designed to enhance trait EI and authoritative parenting practices could provide concrete evidence for effective strategies in fostering safer and healthier digital interactions among adolescents. Finally, given the rapid evolution of AI technologies, ongoing research should regularly update conceptual frameworks and measures, ensuring that studies remain relevant and adequately reflect emerging digital trends and adolescents’ lived experiences.

## 5. Conclusions

This study examined the relationship between adolescents’ AI use and trust in AI, the generational gap with their parents about these aspects, and their association with key emotional and family factors such as trait Emotional Intelligence (trait EI), parenting style, perceived family support, and parental involvement using data from adolescents and their parents in southern Italy.

Despite the data being cross-sectional, self-report, and, at the dyadic level, modest in size, these correlational patterns suggest that a central implication is that expanding AI ecosystems do not diminish the developmental importance of human relationships. If anything, adolescents’ ready access to AI heightens the need for emotionally available, guiding caregivers who help them interpret, regulate, and appropriately calibrate the information provided by AI systems. In attachment terms, we frame this as a family-anchored digital secure base—a warm, containing relational foundation that extends outward to scaffold exploration in online and AI-mediated environments. Strengthening adolescents’ emotional competencies, along with authoritative and dialogic family practices, may therefore represent a promising target for interventions aimed at promoting critical, safe, and developmentally supportive engagement with AI.

## Figures and Tables

**Table 1:** Frequencies about AI usage and trust, and differences between adolescents and parents.

	Adolescents (n = 170) N (%)	Parents (n = 175) N (%)	*χ* ^2^	*V*	95% CI
AI usage: Share personal data			9.36 *	0.17	[0.06; 0.27]
Never	77 (45.5)	85 (50.3)			
Rarely	41 (24.2)	54 (31.9)			
Sometimes	33 (19.5)	15 (8.8)			
Often	12 (7.1)	11 (6.5)			
Very Often	6 (3.5)	4 (2.3)			
AI usage: Seek advice for situations			11.78 *	0.19	[0.08, 0.29]
Never	96 (56.8)	120 (71.0)			
Rarely	36 (21.3)	28 (16.6)			
Sometimes	23 (13.6)	11 (6.5)			
Often	6 (3.6)	8 (4.7)			
Very Often	8 (4.7)	2 (1.2)			
AI usage: seeking general information			7.11	0.14	[0.04, 0.25]
Never	39 (23.1)	58 (34.3)			
Rarely	28 (16.6)	30 (17.8)			
Sometimes	55 (32.5)	48 (28.4)			
Often	31 (18.3)	24 (14.2)			
Very often	16 (9.5)	9 (5.3)			
AI usage: school/work support			20.42 ***	0.24	[0.14, 0.34]
Never	36 (21.3)	69 (40.8)			
Rarely	38 (22.5)	28 (16.6)			
Sometimes	41 (24.3)	43 (25.4)			
Often	33 (19.5)	21 (12.4)			
Very often	21 (12.4)	8 (4.7)			
Trust AI: data security			29.01 ***	0.29	[0.19, 0.38]
Never	27 (16.0)	64 (37.9)			
Rarely	62 (36.7)	37 (21.9)			
Sometimes	55 (32.5)	38 (22.5)			
Often	17 (10.1)	27 (16.0)			
Very often	8 (4.7)	3 (1.8)			
Trust AI: better advice than family			12.68 *	0.19	[0.09, 0.29]
Never	71 (42.0)	86 (50.9)			
Rarely	40 (23.7)	45 (26.6)			
Sometimes	32 (18.9)	31 (18.3)			
Often	15 (8.9)	4 (2.4)			
Very often	11 (6.5)	3 (1.8)			
Trust AI: better advice than friends			6.64	0.14	[0.03, 0.24]
Never	67 (39.9)	80 (47.3)			
Rarely	50 (29.8)	46 (27.2)			
Sometimes	27 (16.1)	32 (18.9)			
Often	14 (8.3)	7 (4.1)			
Very often	10 (6.0)	4 (2.4)			
Trust AI: accuracy of information			11.81 *	0.19	[0.08, 0.29]
Never	20 (11.9)	23 (13.6)			
Rarely	61 (36.3)	39 (23.1)			
Sometimes	52 (31.0)	71 (42.0)			
Often	26 (15.5)	33 (19.5)			
Very often	9 (5.4)	3 (1.8)			
Trust AI: tasks better than humans			9.16	0.16	[0.06, 0.26]
Never	20 (11.9)	23 (13.6)			
Rarely	61 (36.3)	39 (23.1)			
Sometimes	52 (31.0)	71 (42.0)			
Often	26 (15.5)	33 (19.5)			

Note: Number (and % in parentheses). Cramer’s *V* * *p* < 0.05, *** *p* < 0.001.

**Table 2:** Correlational analysis between variables.

	1.	2.	3.	4.	5.	6.
1. Authoritarian						
2. Authoritative	−0.18 * [−0.31, −0.03]					
3. Trait EI	−0.31 *** [−0.43, −0.17]	0.35 *** [0.21, 0.47]				
4. Digital Literacy	−0.07 [−0.21, 0.07]	0.33 ** [0.19, 0.45]	0.09 [−0.01, 0.19]			
5. Parental Involv.	0.19 ** [0.04, 0.33]	0.10 [−0.00, 0.24]	−0.18 *** [−0.28, −0.08]	0.14 ** [0.04, 0.24]		
6. PSS: Family	−0.26 *** [−0.48, −0.03]	0.32 *** [0.06, 0.53]	0.29 *** [0.02, 0.53]	0.33 *** [0.08, 0.54]	0.13 [−0.02, 0.02]	
7. AI usage: per. info	0.23 ** [0.09, 0.37]	0.09 [−0.00, 0.23]	−0.08 [−0.18, 0.02]	0.10 * [−0.00, 0.22]	0.28 *** [0.18, 0.38]	−0.17 * [−0.27, −0.07]
8. AI usage: Behavioral Advice	0.34 *** [0.20, 0.46]	−0.06 [−0.21, 0.00]	−0.17 ** [−0.27, −0.07]	0.09 [−0.01, 0.20]	0.27 *** [0.17, 0.37]	−0.18 * [−0.28, −0.08]
9. AI usage: General Info	0.12 [−0.02, 0.26]	0.07 [−0.00, 0.21]	−0.06 [−0.16, 0.04]	0.22 *** [−12, 0.32]	0.18 *** [0.07, 0.28]	−0.17 * [−0.27, −0.07]
10. AI usage: School/work	0.13 [−0.01, 0.27]	0.07 [−0.07, 0.22]	−0.11 * [−0.21, −0.00]	0.24 *** [0.14, 0.34]	0.21 *** [0.10, 0.31]	−0.19 * [−0.29, −0.08]
11. Tust AI: data safe	0.00 [−0.01, 0.01]	0.07 [−0.07, 0.22]	−0.02 [−0.12, 0.08]	0.20 *** [0.10, 0.30]	0.21 *** [0.10, 0.31]	−0.10 [−0.00, 0.22]
12. Trust AI: better than family	0.30 *** [0.15, 0.42]	0.01 [−0.16, 0.13]	−0.14 ** [−0.24, −0.03]	0.04 [−0.05, 0.15]	0.25 *** [0.15, 0.34]	−0.31 *** [−0.43, −0.17]
13. Trust AI: better than friends	0.30 *** [0.16, 0.43]	0.08 [−0.23, 0.00]	−0.15 ** [−0.25, −0.05]	0.00 [−0.11, 0.10]	0.30 *** [0.20, 0.39]	−0.27 *** [−0.17, −0.27]
14. Trust AI: Accurate Info	0.19 ** [0.04, 0.33]	0.10 [−0.25, 0.00]	−0.08 [−0.19, 0.02]	0.14 ** [0.04, 0.24]	0.22 *** [0.12, 0.32]	−0.22 ** [−0.14, −0.30]
15. Trust AI: works better than humans	0.13 [−0.00, 0.27]	0.07 [−0.22, 0.07]	−0.18 *** [−0.28, −0.08]	0.15 ** [0.05, 0.25]	0.19 *** [0.08, 0.29]	−0.21 ** [−0.43, −0.17]

Note: 1 = Authoritarian parenting; 2 = Authoritative parenting; 3 = Trait EI; 4 = Digital literacy (adolescents); 5 = Parental involvement online; 6 = Perceived family support.AI usage: 7 = share personal data, 8 = seek behavioural advice, 9 = seeking general info, 10 = school/work. Trust in AI: 11 = data safe, 12 = advice > family, 13 = advice > friends, 14 = accurate information, 15 = tasks > humans. Pearson correlations with 95% confidence intervals in brackets. * *p* < 0.05, ** *p* < 0.01, *** *p* < 0.001.

**Table 3:** Cluster profiles—means (SD) of variables for Balanced Users vs. At-Risk Users.

Variables	Cluster 1 (Balanced Users) Mean (SD)	Cluster 2 (At-Risk Users) Mean (SD)
Authoritative parenting	4.22 (0.36)	3.64 (0.80)
Authoritarian parenting	1.93 (0.40)	2.30 (0.55)
Trait EI	4.87 (0.71)	4.30 (0.64)
Parental Involvement	25.83 (4.56)	22.81(8.87)
Perceived family support	24.29 (3.74)	18.31 (5.64)
Digital Literacy (parents)	103.31 (18.24)	98.55 (24.16)
Digital Literacy (adolescents)	100.79 (15.52)	96.72 (18.17)
Screen Time (adolescents)	1.72 (0.88)	2.44 (1.42)
AI usage: Share personal data	1.58 (0.81)	2.63 (1.31)
AI usage: behav. advice	1.23 (0.50)	2.81 (1.33)
AI usage: General Info.	2.10 (1.11)	3.44 (1.32)
Trust AI: better advice than parents	1.45 (0.96)	2.94 (0.96)
Trust AI: better advice than friends	1.58 (0.56)	3.06 (1.18)
Trust AI: Accurate info	2.20 (0.97)	3.38 (1.06)

Note: Centroids (Mean and SD) for the two clusters.

## Data Availability

The raw data supporting the conclusions of this article will be made available by the authors on request.
